# Healthcare Safety Nets during the COVID-19 Pandemic Based on Double Diamond Model: A Concept Analysis

**DOI:** 10.3390/healthcare9081014

**Published:** 2021-08-07

**Authors:** Bom-Mi Park, Hyun-Jung Lee

**Affiliations:** 1 Department of Nursing, Konkuk University, Chungju-si 27478, Korea; spring0317@konkuk.ac.kr; 2 Department of Nursing, The Catholic University of Korea, Seoul St. Mary’s Hospital, Seoul 06591, Korea

**Keywords:** healthcare safety net, safety net, COVID-19 pandemic, double diamond model, concept analysis, healthcare system, health inequality, healthcare accessibility

## Abstract

The purpose of this study was to analyze the concept of the “healthcare safety net” during the COVID-19 pandemic. Walker and Avant’s process of concept analysis was used in this systematic literature review. The attributes of the concept of a healthcare safety net during the COVID-19 pandemic were found to be: (a) capacity, (b) accessibility, (c) health equality, and (d) education. In consideration of these defining criteria, antecedents to the concept were identified as: (a) the COVID-19 pandemic, (b) health inequalities (internal factors and external factors), and (c) healthcare systems (health insurance, screening, protective equipment, medicine, and medical services). Consequences of the concept were: (a) meeting healthcare needs, (b) quality of life, and (c) a decrease in morbidity and mortality. A healthcare safety net is an important concept during the COVID-19 pandemic. In situations like COVID-19, healthcare safety nets are designed to meet safety needs, improve quality of life, and reduce patient turnover and mortality. Based on the results of this study, the development of standardized tools for measuring a healthcare safety net as well as that of policies and systems for resolving a healthcare safety net in the COVID-19 situation is recommended.

## 1. Introduction

### 1.1. Necessity of the Study

COVID-19 has become a pandemic that affects populations globally [1]. In particular, it has had a bigger impact on people with a lower socioeconomic status and people with chronic diseases [1], and it has caused inequality in global society. This is related to a lack of access to medical services, which could mean delays in treatment for COVID-19, potentially causing the disease to affect people more severely and even leading to death [1,2]. In particular, in instances where there is a shortage of hospital beds, healthcare workers, and resources due to an increasing number of patients, the opportunity for everyone to receive equitable treatment can be compromised [3].

Public healthcare enables people to use medical services regardless of region, class, or sector and includes all activities for protecting one’s health [3]. In the COVID-19 era, a healthcare safety net does not merely refer to public hospitals and community health centers [4], it also refers to the idea that patients from vulnerable populations should receive equitable treatment [5]. Additionally, socioeconomic status (SES) has a significant impact on healthcare safety nets [6]. In the case of people with a low SES, it is difficult to secure healthcare resources, which leads to health inequality, and depression, which lowers quality of life [6]. Additionally, people with a low SES feel social isolation more than those with a high SES because of their low financial level and lack of interpersonal resources [6]. People who die from COVID-19 experience serious symptoms, which are eventually related to the healthcare safety net system [7]. This indicates that people with a lower SES have difficulty accessing healthcare and are more susceptible to disease risk than those with a higher SES [7]. To prevent such a situation there should be a strengthening where preventive measures are most needed, which means appropriate measures should take SES into consideration [1].

In the case of Korea, local hospitals, except for tertiary general hospitals, were designated as infection control hospitals to identify the increasing number of confirmed patients and asymptomatic carriers. These infection control hospitals also allowed undocumented immigrants to use screening centers regardless of their visa status [8,9]. Moreover, to establish a healthcare safety net in the COVID-19 era, an equitable and accessible healthcare system was implemented. Additionally, policy-based programs were pursued to increase cooperation among various communities for the benefit of the uninsured and those with subpar coverage for the fees incurred due to referral and medical necessity requirements [7]. Despite such efforts, the global number of confirmed COVID-19 cases had reached 178,837,204 by 24 June 2021 and has only increased over time [10].

The idea of “a safety net” originated in social welfare studies. It refers to providing benefits as a last resort in cases where people are not able to access the minimum level of resources they need due to economic cutback policies [11]. In medicine, a healthcare safety net is defined as legally mandated medical services provided to the uninsured and groups with low social security insurance coverage and includes all institutions and programs that provide such services [12]. Yet despite its importance, there are very few studies related to healthcare safety nets in nursing. The term “safety net” is often used with different interpretations in sociology, medicine, and the arts, and is used in various ways, including “safety net,” “healthcare safety net,” and “social safety net.” Yet, the concept of “a healthcare safety net” remains notably absent in the literature; therefore, the establishment of a new healthcare safety net is needed to equitably address the COVID-19 pandemic and to clearly define what a “healthcare safety net” is [13]. Specifically, the establishment of a healthcare safety net can serve as an important step towards advancing people’s quality of life; therefore, it is necessary to define the concept clearly.

For patient-centered services, service design that can present standardized services based on understanding the experience of the target person and identifying their potential needs is very important [14]. Moreover, as the most commonly used service design at present, the four-step double diamond process of discover, define, develop, and deliver that repeats divergent and convergent thinking is suggested [15]. In this study, we intend to study the medical prospects in the COVID-19 era based on the double diamond model.

A concept explains a phenomenon and includes attributes to provide an idea of what it entails, while providing a new perspective for observing the phenomenon of interest [16]. A concept analysis facilitates communication between the people who use the concept by providing clear meaning through the discovery of terms that are used interchangeably [16]. Currently, a healthcare safety net is being used in various ways; however, it is an increasingly important concept in the COVID-19 era, and a concept analysis that clearly defines it for advanced use in nursing is also needed.

Until now, most of the studies about nursing and COVID-19 have dealt with nurses’ experiences with patient care [17], nurses’ perception about the care needs of patients [18], and nurse reports of stressful situations [17]. It is difficult to find studies that are concept analyses of a healthcare safety net during the pandemic. Accordingly, this study serves to fill a gap in the literature and advance the nursing discipline by enhancing the understanding of the concept of a healthcare safety net and providing basic data to healthcare workers. 

### 1.2. Purpose

The purpose of this study is to clarify the concept of a healthcare safety net during the COVID-19 pandemic based on the double diamond model, and to identify its attributes to create a clearer understanding of what a healthcare safety net is with regards to its antecedents and its consequences.

## 2. Methods

### 2.1. Study Design

This systematic literature review uses a concept analysis that derives the attributes, antecedents, and consequences of healthcare safety nets during the COVID-19 pandemic using the method developed by Walker and Avant [19].

### 2.2. Study Details

To confirm the conceptual attributes and contextual referents, the concept of “healthcare safety net during the COVID-19 pandemic” was included and searches were conducted using “healthcare safety net”, “health safety net”, “safety net”, and “COVID-19.”

**Figure 1:**
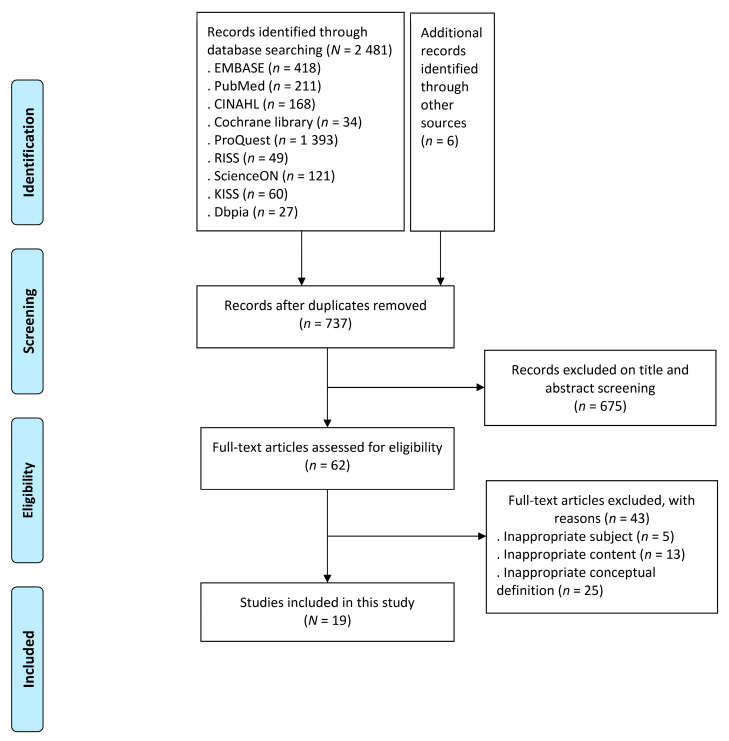
Flow diagram of study selection process.

To identify the conceptual attributes and to confirm how the concept of a healthcare safety net was used and defined in the literature, this study conducted searches in international databases (Embase, PubMed, CINAL, Cochrane, and ProQuest) and Korean databases (RISS, ScienceON, KISS, and Dbpia), published from 1 January 2020 to 30 April 2021. After excluding duplicates, 737 studies remained, of which 675 were excluded based on the title and abstract screening. After reviewing the remaining 62 articles, we eliminated 43 for the following reasons: inappropriate subjects, inappropriate content, and inappropriate conceptual definition for 5, 13, and 25 articles, respectively. Finally, 19 papers were selected (Figure 1).

Contents regarding a healthcare safety net during the COVID-19 pandemic were identified according to the protocol suggested by Walker and Avant [19], and the specific processes were as follows:

- Select the concept.
- Determine the purpose of the concept analysis.
- Identify the range of use of the concept.
- Identify the attributes of the concept.
- Present a model case of the concept.
- Present additional cases of the concept (borderline, contrary, and/or related cases).
- Identify antecedents and consequences of the concept.
- Define empirical referents of the concept.

### 2.3. Study Framework

The double diamond model used in this study was designed from the divergence–convergence model proposed by Béla H. Bánáthy (1996) [15,20]. The model is divided into four steps through two adjacent diamonds, each of which provides a problem and a solution [15]. This model is divided into a continuous divergent–convergence phase as each space has a divergent phase that expands the space and a convergence phase that narrows the space [15]. In the first diamond, the discover and define phases take place, and in the second phase, the develop and deliver phases take place. (1) The discover phase allows us to understand what the problem is. (2) The define phase helps you define in a different way with the insights gathered in the discover phase. (3) The develop phase allows people to come up with different solutions to clearly defined problems. (4) The deliver phase can test and improve various solutions [15].

**Figure 2:**
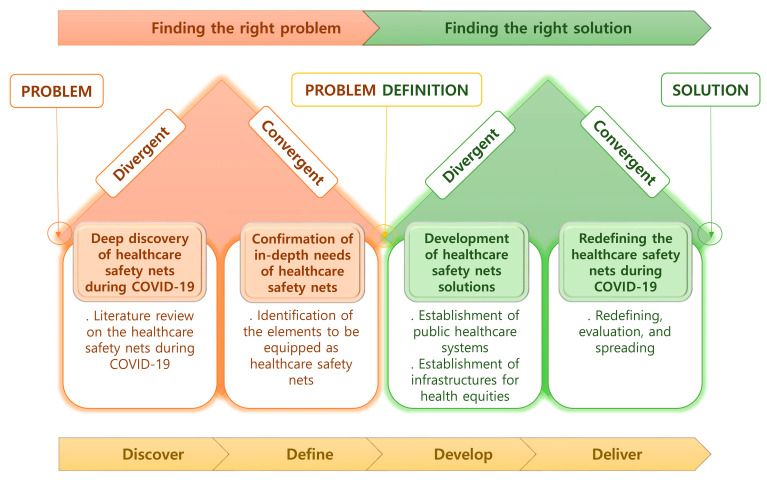
Conceptual framework of a healthcare safety net during the COVID-19 pandemic based on the double diamond model.

In this study, the framework of healthcare safety nets was constructed based on the double diamond model (Figure 2).

(1) Discover: deep discovery of healthcare safety nets during COVID-19
(2) Define: confirmation of in-depth needs of healthcare safety nets
(3) Develop: development of healthcare safety nets solutions
(4) Deliver: redefining the healthcare safety nets during COVID-19

## 3. Results 

### 3.1. Literature Review of Healthcare Safety Nets

#### 3.1.1. Lexical Definition 

“Healthcare safety net” is a compound phrase combining “health” and “safety net.” To examine the lexical definition of the phrase, the definition of “health” was examined first. In previous literature, “health” was defined as “a state in which disease and infirmity are absent” [21]. However, the World Health Organization (WHO) redefined health as “a sound state of physical, mental, and social adaptation, and not merely the absence of disease and infirmity” [22]. Moreover, the literature also defines health as physical and mental well-being, being free from disease and suffering, being healthy in mind and body, and being socially and culturally healthy [16]. A “safety net” is defined as an externally enforced minimum level of health or economic capital [23]. Moreover, a safety net is often viewed as a fail-safe source of healthcare for indigent and uninsured people that everyone in a given population has access to. Further, it provides first-line emergency care and preventative care for vulnerable populations through hospital emergency departments (EDs), emergency medical service (EMS) providers, and public/free clinics [24]. Moreover, COVID-19 is a disease that can infect both humans and animals [25]. A healthcare safety net during the COVID-19 pandemic has been defined as a low-cost option for patient treatment, rather than maintaining the existing high-volume, high-priced healthcare system. This safety net reduces existing gaps in accessibility to specialized treatment and establishes the infrastructure needed to connect people to a healthcare system [6]. Additionally, healthcare safety nets are being used to increase the accessibility and prominence of services provided through telemedicine and video conferencing to prevent the spread of infection [7]. In this sense, the healthcare safety net during the COVID-19 era can be defined as a network that increases healthcare accessibility and equality for all patients. In addition, it creates physical, mental, and social safety through in-person care and telemedicine that enables people to access healthcare anywhere at any time.

#### 3.1.2. Range of Use of the Concept

A typical safety net in other disciplines refers to a social safety net, which is a policy that affords poor people, who otherwise do not have access, a basic standard of living [26]. Especially in the USA, low-income and immigrant populations are not adequately covered by the healthcare system. Specifically, insurance does not always cover treatment for infectious diseases, such as COVID-19. Additionally, the capacity of hospitals to provide medical services to high-risk patients and communities is severely impacted by COVID-19 [27]. In the social welfare sector in Korea, exposure to job crises during the COVID-19 era has led to losses of income as attempts to apply the existing welfare system to those in welfare blind spots have proved insufficient. As a result, greater inequality and inequity threaten the social safety net [28].

A healthcare safety net refers to all systems that address individual and group healthcare needs, and they especially apply to low-income and other vulnerable populations [12]. As the COVID-19 pandemic became more serious, a safety huddle, which is a safety net for nurses, was created, and included nursing cooperation and communication related to patient safety by means of a healthcare safety net. This safety huddle was found to facilitate efficiency, collaboration, and participation between colleagues by communicating about specific operational issues with colleagues and discussing the daily goals of patients, safety issues, and barriers to discharge [29]. While a social safety net in other disciplines focuses on allowing people to live their lives without their jobs, income, or welfare being threatened, a healthcare safety net encompasses healthcare solutions and patient safety. 

### 3.2. Tentative Criteria for and Attributes of Healthcare Safety Nets

The following tentative criteria for and attributes of healthcare safety nets during the COVID-19 pandemic were identified by reviewing various pieces of literature (Table 1 and Table 2). 

#### 3.2.1. Conceptual Definitions of Healthcare Safety Nets in the Literature

1. Supporting the provision of essential care and healthcare systems (A1, A2, A6, A9, A13, A15)
2. Supporting education (A3)
3. Coordinating the medical and public health and policy response (A4, A10, A12)
4. Improving patients’ outcomes related to disease and clinical severity (A5)
5. Improving the rapid screening and treatment of COVID-19 patients (A6, A7, A19)
6. Preserving the capabilities of hospitals and healthcare workers (A6, A18)
7. Providing appropriate personal protective equipment (A7, A19)
8. Use of telemedicine/telephone meetings (A7, A8)
9. Increasing medicine and materials (A11, A16)
10. Addressing social imbalances to improve health and well-being (A14)
11. Monitoring the incidence and imbalance of the disease burden (A17)

#### 3.2.2. Attributes of a Healthcare Safety Net

(1) Capacity

**Figure 3:**
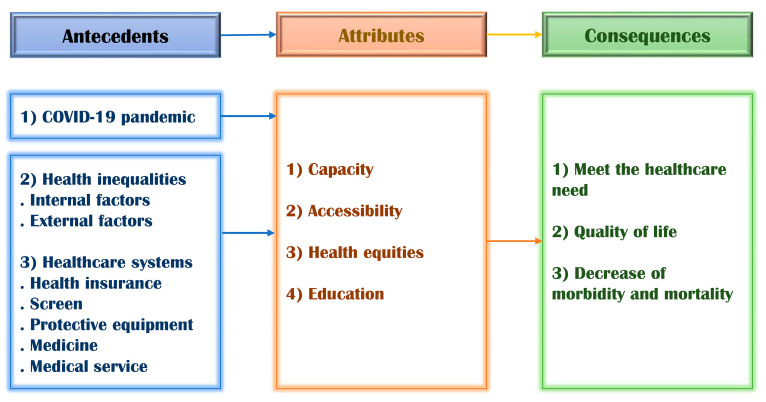
Concept diagram of “a healthcare safety net”.

Capacity refers to the ability to accept and treat patients. The COVID-19 pandemic has allowed for the identification of the following capacity-related issues: hospital capacity (A1), expansion of capacity (A4), improved health systems capacity (A10, A18), and emergency department capacity (A17, A19). Capacity issues arose regarding the space needed for patient isolation, such as in hospitals for treating COVID-19, and the medical staff available to treat and care for COVID-19 patients in medical facilities due to the sudden emergence of the pandemic. Therefore, securing hospitals and facilities and medical staff to work in these places is needed to build an adequate healthcare safety net for instances such as the COVID-19 pandemic (Figure 3).

(2) Accessibility

Accessibility is defined as having easy access to medical facilities (A6, A7, A11, A12) and effective and essential treatment access at these facilities (A4, A11, A15, A19). During the COVID-19 pandemic, priority should be given to COVID-19 treatment to ensure that patients receive treatment in a timely manner (A4). For COVID-19 tests, increased transportation to testing sites is needed (A8). Confirmed COVID-19 patients must be placed in isolation (A5, A7) for follow-up observation by medical staff (A3, A7). Therefore, regional differences in accessibility must be reduced and accessibility to medical facilities must be increased to provide immediate medical services as needed as part of a healthcare safety net during the COVID-19 pandemic.

(3) Health Equality

Health equality refers to the state of having no health disparities between populations [30]. COVID-19 can cause disparities in treatment to arise. Health equality requires that priority cases be prioritized (A1, A2, A4), and when using medical services (A5), all populations must be able to receive adequate treatment (A3). Moreover, health care systems must increase the quality of care and multidisciplinary cooperation to reduce health discrepancies (A9). The highest level of health equality refers to an absence of health inequality, which is the difference in health levels between populations [31]. Therefore, it is important to have a robust healthcare safety net during the COVID-19 pandemic to ensure that discrepancies in treatment among different populations do not arise.

(4) Education

During the COVID-19 pandemic, systematic infection control education, along with job manual development and educational programs have been needed [8]. Currently, previous studies are providing formal instructions about COVID-19 transmission methods, symptoms that may appear, treatment, and prevention methods (A7). Education about these topics should be conducted in various medical facilities via telephone and video (A9). Moreover, increasing public health education can reduce public health disparities (A14). Additionally, education about preventive measures can minimize the risks faced by all medical staff during the COVID-19 situation (A7). Education about prevention and treatment is needed to create an adequate healthcare safety net during the COVID-19 pandemic.

### 3.3. Construction of a Model Case of the Concept

The model case of a concept refers to a case that includes all the key attributes of that concept [19]. In this study, the model case is presented based on the four major attributes of a COVID-19 healthcare safety net. The attributes are indicated with their numbers in parentheses.

Johnson, who has amyotrophic lateral sclerosis, is a 70-year-old male retiree living with his family in the countryside. Despite the difficulty of leaving home due to a recent increase in confirmed COVID-19 cases, he made an appointment by telephone for treatment for amyotrophic lateral sclerosis at a hospital in a large city (2). There were live TV broadcasts about the global situation and precautions regarding COVID-19, warning people to refrain from going out unnecessarily (4). Nevertheless, Johnson needed to receive treatment despite the COVID-19 situation. Fortunately, he was able to receive treatment from a skilled physician (1). Despite living in the countryside, he was able to receive his treatment without much difficulty since there was a direct bus route to a large hospital (2). Because of the COVID-19 pandemic, he had his temperature checked on the first floor and underwent a COVID-19 test upon arrival at the hospital. COVID-19 tests were previously expensive, but because of a recent increase in the number of confirmed cases, anyone who wanted to be tested could get tested for free, even without any symptoms of a fever (3). Fortunately, the COVID-19 test result was negative, and he was admitted (1) to a hospital ward. There, he received inpatient education consisting of information about COVID-19-related precautions and prevention from the nurse in charge (4). Additionally, Johnson was able to receive inpatient care (1), as planned, for amyotrophic lateral sclerosis. 

### 3.4. Additional Cases of the Concept 

#### 3.4.1. Borderline Case

A borderline case is a case that includes some, but not all, of the key attributes of the concept presented in the model case [19]. In this study, the borderline case addresses the issues of capacity, accessibility, and education. 

Julia, a 25-year-old nurse, was working in a general ward, but was transferred to the COVID-19 infection ward that was newly created in 2020 in response to the COVID-19 pandemic. The COVID-19 unit consisted of a single ward with 20 beds (1). All patients confirmed to have COVID-19 were admitted to the newly established infection ward despite the fact that there was still a shortage of nurses and medical staff (1). When a patient was admitted, education was provided, not only about the general procedure in the ward, but also about COVID-19 (4). If admitted to a private room, the patient could not leave the room and was required to undergo weekly COVID-19 testing (2). The physician in charge applied examination and nursing procedures that required minimal human resources and was required to provide patient care in a designated room while wearing level D protective equipment in accordance with COVID-19 guidelines (2). The medical staff provided patient care while being equipped with a personal radio and there was no separate pathway to the outside. All examinations and treatments were administered simultaneously to minimize contact with the patient after entering the room, while information and updates about COVID-19 were provided constantly to the patients to alleviate their anxiety (4). Patients who had three or more consecutive negative test results were informed about having to prepare for discharge and were educated on droplet infection prevention methods, mask wearing methods, hand washing methods, and social distancing procedures (4).

#### 3.4.2. Contrary Case

This type of case does not display attributes of the concept, but presents attributes that are contrary to it [19]. The presentation of a contrary case allows the attributes of the concept being explained by the researcher to be better understood and clarified. This case did not include any attributes of a COVID-19 healthcare safety net; thus, it can be considered a contrary one.

Eighty-year-old Nixon lived in a nursing home with a caregiver but no guardian; his family received confirmation that he had COVID-19. The medical staff at the nursing home attempted to transfer Nixon to a designated COVID-19 hospital; however, the nearby hospital was already full and could not admit any more patients. However, because of the health risks to other patients at the nursing home, the nursing home instructed the family that they had to transfer Nixon to another hospital or take him home for isolation. Admission to nearby designated infection hospitals was difficult, and admission to other hospitals required a COVID-19 test before admission, and getting tested was a challenge. The family wanted Nixon to stay at the nursing home since they could not take care of him, but the nursing home insisted that Nixon had to be discharged immediately. 

#### 3.4.3. Related Case

A related case is one that does not have the key attributes of the concept [19]. While there may be some similarities between the concepts, there are differences in the attributes being analyzed, and thus, it has a different meaning in the concept analysis. 

Lily, in her 30s, is an end-of-life care patient who was diagnosed with leukemia and had several rounds of anti-cancer therapy. Due to the current COVID-19 pandemic, patient visits were impossible in most cases. However, because Lily was designated as an end-of-life care patient, she was allowed up to four visitors per day. Moreover, while there were no single-occupancy rooms due to COVID-19, she was able to use a room by herself due to her designation as an end-of-life care patient. The medical staff explained to Lily the need for restricting the number of visitors due to the COVID-19 pandemic.

### 3.5. Identification of Antecedents and Consequences of a Healthcare Safety Net

#### 3.5.1. Antecedents

Antecedents refer to additional conditions or events before the occurrence of the concept [19]. Based on the literature review, the following antecedents of a healthcare safety net during the COVID-19 pandemic were identified (see Table 2 for more details).

1. COVID-19 pandemic
2. Health inequality
  - Internal factors
  - External factors
3. Healthcare systems
  - Health insurance
  - Screening
  - Protective equipment
  - Medicine
  - Medical services

#### 3.5.2. Consequences

Consequences refer to additional conditions or events after the occurrence of the concept [19]. The following consequences of a healthcare safety net during the COVID-19 pandemic were identified.

1. Meet the healthcare need
2. Quality of life
3. Decrease in morbidity and mortality

### 3.6. Empirical Criterion

Empirical criterion is the final stage of concept analysis, and it demonstrates the actual existence of the attributes of the concept in the field. Empirical criterion is not a tool for measuring the concept itself. Instead, it is a method that can measure or recognize defined characteristics or attributes [19]. There were no tools for measuring healthcare safety nets during the COVID-19 pandemic in the articles studied. Therefore, the empirical criterion for healthcare safety nets during the COVID-19 pandemic could be identified using the characteristics identified in this study [18]. 

COVID-19 forced healthcare professionals, healthcare systems, scientists, and policy makers to resolve social inequalities to improve health and well-being [32]. Moreover, healthcare safety nets during the COVID-19 pandemic attempted to increase health equality by eliminating barriers and adapting to the newly improved healthcare system [33]. The major characteristics of a healthcare safety net during the COVID-19 pandemic were reduced morbidity and mortality (A1, A15), where, if all patients could receive hospital treatment within a healthcare safety net, the mortality rate due to COVID-19 would decrease [31]. Additional characteristics described included health systems capacity (A6), hospital capacity (A1), access to testing (A4), assessing the efficacy of vaccines (A11), flexibility in the location of service (A6), quality care (A9), healthcare resource allocation (A10), and public health education (A14). Securing the safety net and protecting public health during a pandemic requires: (1) improving prompt testing and treatment of patients with COVID-19, (2) mitigating the strain on the healthcare system and preserving the capacity of hospitals and healthcare practitioners, and (3) limiting the transmission of COVID-19 [7]. Additionally, several reports reflect the disproportionate burden of COVID-19 infection and its related morbidity and mortality among patients [34,35]. 

## 4. Discussion

To control COVID-19 and prevent unnecessary suffering and economic loss, group behavior must be improved [36] and a clear definition of a healthcare safety net must be established [37]. This study used the concept analysis developed by Walker and Avant (2005) [19] and the double diamond model (1996) [15] to analyze the concept of the healthcare safety net during COVID-19. Capacity, accessibility, health equality, and education were identified as the attributes of this analysis. 

Healthcare safety is a broad concept, and a net is any system that provides an opportunity to meet the needs of individuals or households facing difficulties. While the concept includes all people, low-income and poor groups are a narrow concept within it [38]. A healthcare safety net is very important for public safety since it is the sole provider of first-line emergency care, as well as routine healthcare through hospital EDs, EMS providers, and public/free clinics [24]. In particular, the ED is where the occurrence of disease burden and imbalances can be monitored, and targeted and culturally appropriate responses implemented [35]. Therefore, to increase the capacity of a healthcare safety net, support for physician practices, hospitals, and healthcare systems [39], expansion of treatment capabilities, and maintenance of the capacity of treating hospitals [40] must be ensured. 

Given that COVID-19 is a global pandemic, healthcare services need to be highly accessible [41]. However, socioeconomic disparities have only worsened during the COVID-19 pandemic [32], with difficulties in accessing telemedicine for low-income people and those with language limitations emerging as increasingly relevant issues [42]. Good access to healthcare services can help expedite detection and treatment of COVID-19 patients [32,41] and reduce the mortality rate [43]. Accordingly, to increase the accessibility of a healthcare safety net, the accessibility of essential healthcare must be ensured [33], including COVID-19 testing [40], access to healthcare services [41], telemedicine services [33], and vaccinations [43]. 

Health equity implies the absence of unjust and preventable differences in health between groups of people [31]. However, a lack of personal protective equipment, limited staff to screen for COVID-19 [40], language barriers, insufficient data plans, and legal barriers in accessing technology or the Internet [44] have caused health inequalities. Moreover, health disparity is associated with an increased risk of serious diseases due to COVID-19 infection [45]. Therefore, people must be able to receive essential treatment [37] regardless of race and ethnicity [32], insurance coverage, or financial background to ensure that healthcare inequalities do not occur during the COVID-19 pandemic. In particular, public policy responses at government levels must call [46] for the expansion of essential healthcare [38], equitable allocation of resources [46], and balance [42]. 

A healthcare safety net encompasses both first-line emergency care and routine healthcare [24]. In particular, COVID-19 has forced healthcare professionals, healthcare systems, scientists, and policy makers to resolve social inequalities to improve the health and well-being of people [32]. However, to prolong this effect, treatment and public health education are needed [32]. Additionally, education on the rapid utilization of health information technology to coordinate healthcare and public health responses to COVID-19 is needed [40]. Educational programs help nurses better care for their patients, increase their confidence, and ultimately improve the quality of patient care [47]. Government and health systems are providing education to those affected by COVID-19 for psychological and social cohesion, as well as providing updates on the discovery and development of the latest pandemic, improving nurses’ abilities to care for patients with COVID-19 [48]. Additionally, nurses contribute to providing a better working environment by providing emotional support through education to employees who are concerned about being infected with the virus themselves [49]. Therefore, a healthcare education system that can enhance the level of knowledge about COVID-19, actively practice prevention activities [50], and increase patient participation must be implemented [44]. 

Nursing personnel account for nearly 50% of the global health workforce, providing nursing in hospitals and long-term care facilities, and they are at the forefront of the fight against the spread of the pandemic [51]. As COVID-19 spreads worldwide, nurses are disproportionately vulnerable to COVID-19 and are physically and mentally exhausted as a result of taking care of patients infected with COVID-19 [52]. Therefore, a clear definition of a health safety net in nursing is needed [52].

Most countries are struggling with COVID-19 because they have not listened to public health advice after epidemics, like the last outbreak of SARS [53]. Additionally, differences in socioeconomic status and health inequality have become clearer, while the concept of an appropriate healthcare safety net is not clear [53]. Public hospitals, which played a major role in Korea’s COVID-19 situation, ensured healthcare access for patients, and public hospitals designated as COVID-19 exclusive hospitals to secure negative pressure isolation beds for intensively treated infectious patients [54]. Anyone with no symptoms such as fever, headache, or difficulty breathing was provided with free COVID-19 preemptive tests so that asymptomatic patients could be detected and treated in advance [54]. It was possible to receive COVID-19 treatment such as treatment, nursing, counseling, examination, medication, and monitoring at the living treatment center [54]. It is thought that public hospitals include all the attributes of a healthcare safety net.

To protect healthcare staff from the highly contagious COVID-19 virus and to protect other patients visiting hospitals and public health centers, a temporary clinic has been established outside the building to collect samples or drive-thru so that testing can be done without getting out of the car [55]. Through intensive efforts from all government ministries led by the Korea Centers for Disease Control and Prevention (KCDC), and active participation of the healthcare community and citizens, the Republic of Korea is carrying out an effective quarantine project [55]. The KCDC is receiving support from the public by quickly and transparently delivering accurate information about COVID-19 to the public [55]. However, early education needs to be strengthened [55]. 

In order to solve the health equity problems, an urgent response from the downstream and countermeasures for contributing factors from the upstream or middle are required at the same time [56]. For example, if measures at the individual level are generally referred to as downstream-level approaches to reduce the effect of the results on a population group that shows discriminatory outcomes such as health behaviors or health levels, it is likely that measures that contribute to the health gap measures at the institutional level, such as socioeconomic conditions like employment, education, and income disparity, are upstream approaches [56].This is considered to be related to the internal and external factors of health inequality suggested as antecedent factors of the healthcare safety net in this study. It is believed that the effectiveness of the healthcare safety net can be increased by lowering the factors of health inequality presented in this study. In the response stage of a global pandemic, while focusing on urgent responses to immediate burdens such as disaster aid and unemployment benefits, in preparation and recovery, measures related to social health determinants should be emphasized [57]. For example, it will be necessary to invest more in equity in areas such as the establishment of a public health and healthcare system, basic economic security, guaranteed access to digital infrastructure, establishment of a system to comply with quarantine rules, and safe and equitable social infrastructure [57]. 

Populations socially and economically deprived due to COVID-19 are vulnerable to epidemics [7], and marginalized populations who have experienced past epidemics and recent natural disasters will see health inequality [58]. Therefore, it will be possible to reduce the gap between SES and health inequality from future infectious diseases and natural disasters by taking economic and social protective measures for those who are vulnerable to COVID-19 and suffer from health inequality. In particular, Korea has many COVID-19-positive cases in the workplace [59]. Therefore, workers of large companies can work from home, but workers of small and medium-sized enterprises are vulnerable to COVID-19 as they are exposed to the risk of unemployment due to COVID-19 [60]. Accordingly, Korea is making efforts to prevent the spread of group infection by utilizing social distancing, flexible working hours, early identification of suspected infected workers, and disinfection of the workplace to prevent group infections occurring at the workplace [59]. 

Walker and Avant (2005) [19] and the double diamond model (1996) [15] can model the exact use process of the concept of healthcare safety nets and provide healthcare safety nets redefined in the COVID-19 situation through appropriate processes for each step. This process allows us to identify and visualize the potential needs of the people in healthcare safety nets. The healthcare safety net presented in this study is designed to be applicable to all citizens, and through this healthcare safety net, all citizens can receive healthcare services in the COVID-19 situation. Furthermore, it is intended to suggest a direction to improve their quality of life and reduce mortality. Recognizing that it is difficult to end COVID-19, we need to devise a strategy to combat COVID-19 by mobilizing all resources, including the healthcare safety net [60]. 

In this study, it is thought that the function of the healthcare safety net can be increased by reducing health inequalities and improving the healthcare systems, which are the prerequisites for the healthcare safety net in the era of COVID-19. Additionally, if the functions of capacity, accessibility, health equities, and education are expanded through cooperation between the government, public institutions, and private institutions, it is shown that the rate of infected patients from COVID-19 will decrease. The severity of the outbreak across different countries varies significantly due to several factors, such as timeliness and strength of state interventions, country healthcare readiness, and socioeconomic considerations [61,62]. Nevertheless, the ultimate outcome of the healthcare safety net in the context of COVID-19 is meeting healthcare needs, increasing quality of life, and decreasing morbidity and mortality, which apply not only to South Korea, but also to other countries suffering from COVID-19. 

This study presented antecedents, attributes, and consequences based on previous studies on COVID-19. However, a healthcare safety net during the COVID-19 pandemic involves a combination of factors, which cannot be explained via a straightforward model. This study was significant in that it presented the concept of a healthcare safety net during the COVID-19 pandemic by analyzing the points presented in studies on COVID-19 published in the past 18 months. The following recommendations are made. First, active studies are needed that recount the pandemic from the perspective of nurses. These studies must include the required attributes of a healthcare safety net based on the findings in this study. Since studies on a healthcare safety net are scarce in nursing, additional studies on healthcare safety nets from the nursing perspective are needed. Second, a healthcare safety net during the COVID-19 pandemic affects and is affected by various antecedents. In particular, because there are limitations as to how much an individual can do, government policies or studies related to institutional improvement are needed. Third, it is necessary to develop assessment tools, including for measuring the antecedents and attributes of a healthcare safety net during the COVID-19 pandemic, and programs that can enhance a healthcare safety net.

## 5. Conclusions

This study used the method by Walker and Avant [19] to conduct a concept analysis to identify the meaning and attributes of a healthcare safety net during the COVID-19 pandemic. A healthcare safety net during the COVID-19 pandemic is intended to satisfy safety needs, enhance quality of life, and reduce morbidity and mortality rates. Through the findings in this study, antecedents of a healthcare safety net during the COVID-19 pandemic were identified to be the COVID-19 pandemic, health inequalities (internal and external factors), and the healthcare system (health insurance, screening, protective equipment, medicine, and medical services). Meanwhile, the attributes were identified as capacity, accessibility, health equality, and education. Additionally, the four-step configuration of the double diamond model (1996) was able to identify and materialize healthcare safety nets that were not clearly revealed during the divergent and convergent process. Accordingly, since healthcare safety nets during the COVID-19 pandemic are currently linked directly with the health of everyone around the world, it is necessary to recognize this concept more clearly and use it more accurately. In addition, at a time when the world is struggling with COVID-19, all countries should share a healthcare safety net response system to prevent possible infectious diseases and think about ways to improve the quality of life for the people.

This study establishes the concept of a healthcare safety net during the COVID-19 pandemic and provides a clearer understanding of this concept. Accordingly, the results of this study can prompt further research related to the development of policies and systems to create a healthcare safety net that adequately addresses the COVID-19 pandemic, as well as the development of standardized tools to measure a healthcare safety net.

## Figures and Tables

**Table 1:** A literature review of the healthcare safety net.

	Author (Year)	Conceptual Definition
A1	Bryan A.F. (2021)	Supporting individual beneficiaries and surgical and physician practices, hospitals, and healthcare systems
A2	Chatterjee P. (2020)	Providing essential care to patients regardless of their insurance coverage, financial circumstances, or immigration status
A3	Bauer K.W. (2021)	Improving emergency food access, access to the program, program’s data systems, and supporting early childcare and education
A4	Sadasivaiah S. (2020)	Coordinating the medical and public health response to COVID-19
A5	Hsu H.E. (2020)	Improving patient outcomes related to disease and clinical severity, such as age, race/ethnicity, homelessness, and underlying medical conditions, and easing the burden on the health care system from COVID-19
A6	Bachireddy C. (2020)	Improving the rapid screening and treatment of COVID-19 patients, easing the burden on the healthcare system, preserving the capabilities of hospitals and healthcare workers
A7	Napoleon S.C. (2020)	Screening patients for possible exposures and/or symptoms of COVID-19, providing appropriate personal protective equipment to all staff when possible, and using telemedicine/telephone meetings
A8	Bhaskar S. (2020)	Integral part of the public health response during COVID-19, and telemedicine serves as a safety net in the public health response
A9	Velázquez P.P. (2020)	Ensuring a timely, equitable, safe, and appropriate approach to quality care for all patients
A10	Bambra C. (2020)	Correcting public policy responses such as social protection and public service expansion and pursuit of green inclusive growth strategies
A11	Dhanda S. (2020)	Increasing the vaccination rate of the population
A12	Cheng T.L. (2020)	Public health and safety net infrastructure
A13	Lau J. (2020)	Keeping staff and patients safe, managing limited resources, maintaining access to treatment, and proactively meeting the needs of the most vulnerable residents
A14	Lopez L. (2021)	Providing clinicians, healthcare systems, scientists, and policy makers the opportunity to address social imbalances to improve health and well-being
A15	Anand P. (2020)	Providing coverage to low-income individuals who are uninsured, or not eligible for other insurance options
A16	Zumla A. (2020)	Creating of Biobank and access to biological materials
A17	Misa N.Y. (2020)	Monitoring the incidence and imbalance of disease
A18	Blumenthal D. (2020)	Improving the capacity for collective action to protect the public’s health
A19	Kendzerska T. (2021)	Optimizing the distribution of personal protective equipment and staff to urgently treat individuals with COVID-19, and protecting patients

**Table 2:** Antecedents, attributes, and consequences of a healthcare safety net.

Dimension	Sub-Dimension		Key Findings in Reviewed Literature
antecedents	COVID-19 pandemic		Shock/shutdown/closure/crisis/financial downturn (A1), closed/motivated rapid (A3), exposure (A5, A10, A19), skyscraping death (A8), lockdown/inequalities in prevalence and mortality rates (A10), crisis (A12, A19), disaster (A13), invasion (A15), disproportionate burden and mortality (A17)
	Health inequalities	Internal factors	Disproportionately/postpone/treat/loss/bankruptcy (A1), gap (A3, A6), lack of consistent/restriction (A3), rely on care/limited health literacy (A4), barrier/limit (A4, A6, A8, A9, A13, A17, A19), race and ethnic health disparities (A5, A14, A17), insufficient or no healthcare insurance (A10)
		External factors	Vulnerably/excludes (A3), minority/homelessness/illness/clinical severity (A5), higher risk/lack of personal protective/limited staff to screen/limited resources/limited laboratory services (A7), inequalities in morbidity and mortality rates/unequal experience/deprived area/disadvantage (A10), educational and technological disparities (A12), lack of staff (A13), minority (A14)
	Health care systems	Health insurance	Support of individual beneficiaries/surgical and physician practices/hospitals and healthcare systems (A1), health insurance (A1, A2, A9, A12, A13, A15), coverage (A1, A2, A6, A13, A14, A15, A18)
		Screen	Monitoring/services (A3), expanding testing (A4), screening procedure (A7), screening patients (A7, A14) Detection of outbreaks, disparities in disease burden, and surveillance (A17)
		Protective equipment	Medical equipment (A6), protective equipment (A7), protect/multisystem (A12), protect equipment (A19)
		Medicine	Vaccine and medicines (A11), medications (A19)
		Medical service	Authority/treatment (A6), telehealth services (A6, A13), health care services/improve infection control efforts (A7, A19), network/collaboration (A8), care delivery/prioritize timely (A9), delivery system (A13), share (A13, A19)
Attributes	Capacity		Flex/hospital capacity (A1), maximize/expand/equity (A4), preserving/rapidly expanding/supporting care (A6), improved health systems capacity (A6, A10, A18), hotline (A13), emergency department (A17, A19)
	Accessibility		Baseline system (A2), prioritized (A3, A7), access to test (A4), urgent care (A4, A19), utilization (A5), ease/flexibility in location of service (A6), access to healthcare service/relationship (A7), contact (A7,12), telehealth implementation/telemedicine/moved to (A8, A19), conveniently/assessing the efficacy of vaccine (A11), access to primary care (A13, A15)
	Health equalities		Essential care (A1, A2), vital (A2), primary site of care (A2, A4, A19), normal/benefit (A3), control/eligible for (A6), collaboration/multidisciplinary/quality care (A9), quality/healthcare resource allocation (A10), balancing (A11), triaged (A19)
	Education		Recommended (A7), instruction (A9), public health education (A14)
Consequences	Meet the health care need		Avoid prolonged hospitalization (A5), improved healthcare access/treatment quality (A10), cooperation/healthy individuals (A11), management/connection (A13), paradigm shift (A16), consistent(A19)
	Quality of life		Promote health (A4), adoption (A13), health and well-being (A14), Quality of life (A16)
	Decrease morbidity and mortality		Life expectancy (A5), reducing morbidity and mortality (A11, A12, A16), prevent end organ damage/governing protective or deleterious outcomes (A16)

## Data Availability

Not applicable.
